# Modalities of sequential human robot collaboration trigger different modifications of trunk oscillations

**DOI:** 10.3389/fnbot.2023.1183164

**Published:** 2023-06-22

**Authors:** Simone Ranaldi, Daniele Bibbo, Giovanni Corvini, Maurizio Schmid, Silvia Conforto

**Affiliations:** Department of Industrial, Electronic and Mechanical Engineering, Roma Tre University, Rome, Italy

**Keywords:** collaborative robotics, ergonomics, inertial measurement unit, biomechanical risk, movement analysis

## Abstract

**Introduction:**

Human robot collaboration is quickly gaining importance in the robotics and ergonomics fields due to its ability to reduce biomechanical risk on the human operator while increasing task efficiency. The performance of the collaboration is typically managed by the introduction of complex algorithms in the robot control schemes to ensure optimality of its behavior; however, a set of tools for characterizing the response of the human operator to the movement of the robot has yet to be developed.

**Methods:**

Trunk acceleration was measured and used to define descriptive metrics during various human robot collaboration strategies. Recurrence quantification analysis was used to build a compact description of trunk oscillations.

**Results and discussion:**

The results show that a thorough description can be easily developed using such methods; moreover, the obtained values highlight that, when designing strategies for human robot collaboration, ensuring that the subject maintains control of the rhythm of the task allows to maximize comfort in task execution, without affecting efficiency.

## 1. Introduction

In industrial environments, robots have been used increasingly to help or replace workers in repetitive and demanding tasks. The robot evolution has allowed a closer interaction to allow what is nowadays defined as collaboration or cooperation. The collaboration between humans and robots (HRC) has recently become one of the main topics in the scientific literature about robotics (Ajoudani et al., 2018, 2020; Matheson et al., 2019). In particular, the technologies behind HRC have been developed aiming to allow the inclusion of collaborative robots in a variety of different workplaces to help human operators executing work-related tasks in a safer and more efficient way. Considering this, a complete platform for risk assessment during HRC can be of crucial importance to implement effective solutions in helping to improve performances and safety of human operators. While most of the focus has been historically put on the assessment of the risk associated with the robot directly injuring the operator (Inam et al., 2018), recently the ergonomics implications of different collaboration modalities are being investigated as an additional risk factor (Lorenzini et al., 2019; Realyvásquez-Vargas et al., 2019).

Different ways of measuring or controlling ergonomics-related parameters during work activities have been developed during the recent years from methods for the simulation of ergonomic parameters (Greco et al., 2020) to wearable solutions for real-time monitoring (Cheng et al., 2013; Ranavolo et al., 2018; Fortini et al., 2020). In this scenario, wearable technologies represent an important solution due to their straightforward applicability for real-time monitoring of workers' ergonomic assessment (Tsao et al., 2019; Meltzer et al., 2020) and ergonomics training (Lind et al., 2020).

Among the different variables that can be measured as indicators of ergonomic performance and biomechanical risk when standing, trunk oscillations, which directly reflect information about postural sway (Reynard et al., 2019) represent valuable variables that can be associated with fatigue emergence, and, as a result, risk increases (Dupuis et al., 2021). Postural sway has been frequently used for the characterization of ergonomics (Padula and Coury, 2003; Leban et al., 2017; Arippa et al., 2022). Stability and regularity of trunk oscillations is of key importance for the characterization of the motor performance during a biomechanical task (Granata and England, 2006); following this perspective, different methodological approaches, such as the extraction of the maximum Lyapunov exponents (Granata and England, 2006) or the computation of the mutual information (Anagnostou, 2022) have been introduced to capture stability- or regularity-related indicators from trunk movements or from the Center of Pressure (CoP) displacement; given the rather complex nature of the mechanisms of controlling balance while standing, which involves nonlinear effects. Recurrence quantification analysis (RQA) is a convenient method able to capture the deterministic dynamics of body sway (Webber and Zbilut, 1994; Riley et al., 1999). RQA has been shown to reliably capture characteristics of stability and regularity when motor tasks are added to upright stance maintenance (Huang and Hwang, 2013).

In line with the presented literature, we thus used parameters extracted from trunk acceleration data to capture differences in whole body movements across different modalities of human robot collaboration in sequential (*i.e*., in which the two parts do not act on the object at the same time) tasks performed while standing. The extraction of traditional parameters from such data was accompanied with the processing of parameters coming from RQA and by determining summary measures of duration within regions of different movement efforts. In analyzing such parameters, results will be interpreted aiming at describing the different motor control implications of collaborating with a robot in three different sequential modalities, characterized by different rhythms and with different levels of robot intervention. RQA integrates the information coming from traditional statistical indicators of acceleration, while the latter ones are typically appropriate for differentiating the risk associated with trunk movements, and they may be less prone to capture differences when tasks are more complex and possibly unconstrained.

The results presented here are analyzed in terms of their capability of identifying multiple feature sets from three different collaborative scenarios; such a description is intended as aiming to build a compact description, suitable for real-time implementations, that can be exploited both for monitoring workers' physical status during a work shift, as well as for adapting robot behavior with human-based optimization parameters.

## 2. Methods

### 2.1. Experimental set-up

**Figure 1:**
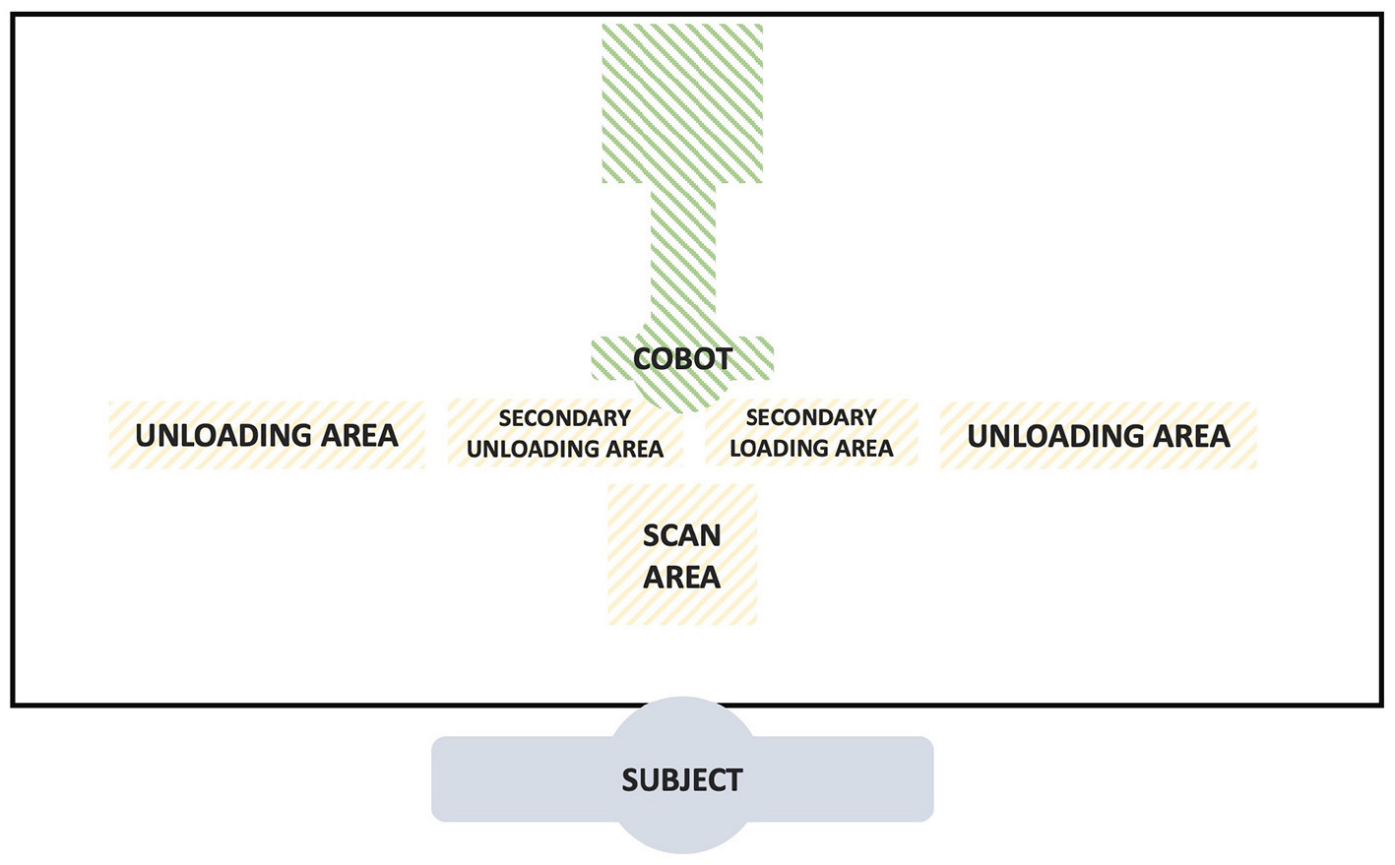
Representation of the workbench used in the experiment (view from above).

The experimental set-up was designed using a work station table, with two main areas at specific distances from the trunk of the worker, one *loading* area to their right hand and one *unloading* area to the left side; between these, a small *scanning* area was designed, in order to simulate a typical cashier work station. Each participant was asked to stand in front of the table, while a collaborative robot (Franka Emika Panda, Munchen, Germany) was fixed to the opposite side of the table with respect to the subject. A schematic representation of the workbench related to the experiment is given in Figure 1.

Participants were asked to cyclically reproduce a working task, consisting of those as follows: (1) taking a 1 kg salt package, marked with a QR code on a face, from the *loading* area, (2) searching for the QR code, (3) simulating a scan in the scanning area, and finally, (4) placing the package into the *unloading* area. Subjects were asked to take the package from the loading area with their right hand and to place it into the *unloading* area with their left hand. No other specific indications were given to subjects to perform the task. This process was then repeated multiple times with packages having the QR code placed in different positions, for 5 min. This trial was then repeated in four different modalities: alone (i.e., named *full*) and in three different collaboration modalities, described in the following section. The modality sequence was assigned randomly to each participant.

In total, 13 healthy subjects participated to the experiment (age 29 ± 3 years old, height 180 ± 9 cm, weight 76.5 ± 6.0 kg, and median ± IQR). Linear acceleration data in the plane approximately normal to gravity were recorded through an inertial measurement unit (Shimmer 3. Shimmer Sensing, Dublin, Ireland), using a sampling rate fs = 100 Hz. To reduce movement artifacts, the sensor was fixed to the subject's back (approximately L4-L5 level) through an elastic band and adhesive tape. Acceleration data components were aligned to the trunk medio-lateral (ML) and antero-posterior (AP) axes, respectively.

### 2.2. Robot collaboration modalities

The collaborative robot was programmed to interact with the task in the following modalities:

- *half robot touch*. The robot takes the package from the *loading* area and waits for a touch by the operator. After the touch command, the robot brings the package close to the *scanning* area (*secondary loading area*) in front of the subject, who grabs the package, and then scans it on the specific area. The unloading phase is carried out by the subject as in the *full* trial when the robot is not used. In this modality, the cycle rhythm is given by the operator.
- *half robot*. Similar to the *half robot touch* modality, with the only difference that the robot moves independently from the touch input by the operator. In this modality, the velocity at which the robot operates is fixed, and all the operations are carried out regardless of the subject's natural rhythm.
- *full robot*. Similar to the *half robot* modality. After the scanning, the package is placed into an area (*secondary unloading area*) closer to the scanner, and the robot moves it to the actual *unloading* area.

In all the collaboration modalities, speed, and periodicity of the robot operations were set so that the subject was able to complete the scanning task in most of the repetitions. This results in waiting phases of different lengths for the different modalities.

### 2.3. Data processing

#### 2.3.1. Data conditioning

Acceleration components were processed as indicators of trunk medio-lateral and antero-posterior kinematics. All the data were low-pass filtered (5 Hz, 3^*rd*^ order Butterworth filter) prior to any analysis. The first and last cycles of any trial were excluded after visual inspection in order to get only the cycles that were executed at steady conditions. In total, at least 4 min were analyzed from each recording.

From the raw data, the maximum absolute value and the RMS were calculated for each trial, both for the radial component and for AP and ML components.

#### 2.3.2. Acceleration phases definition

**Figure 2:**
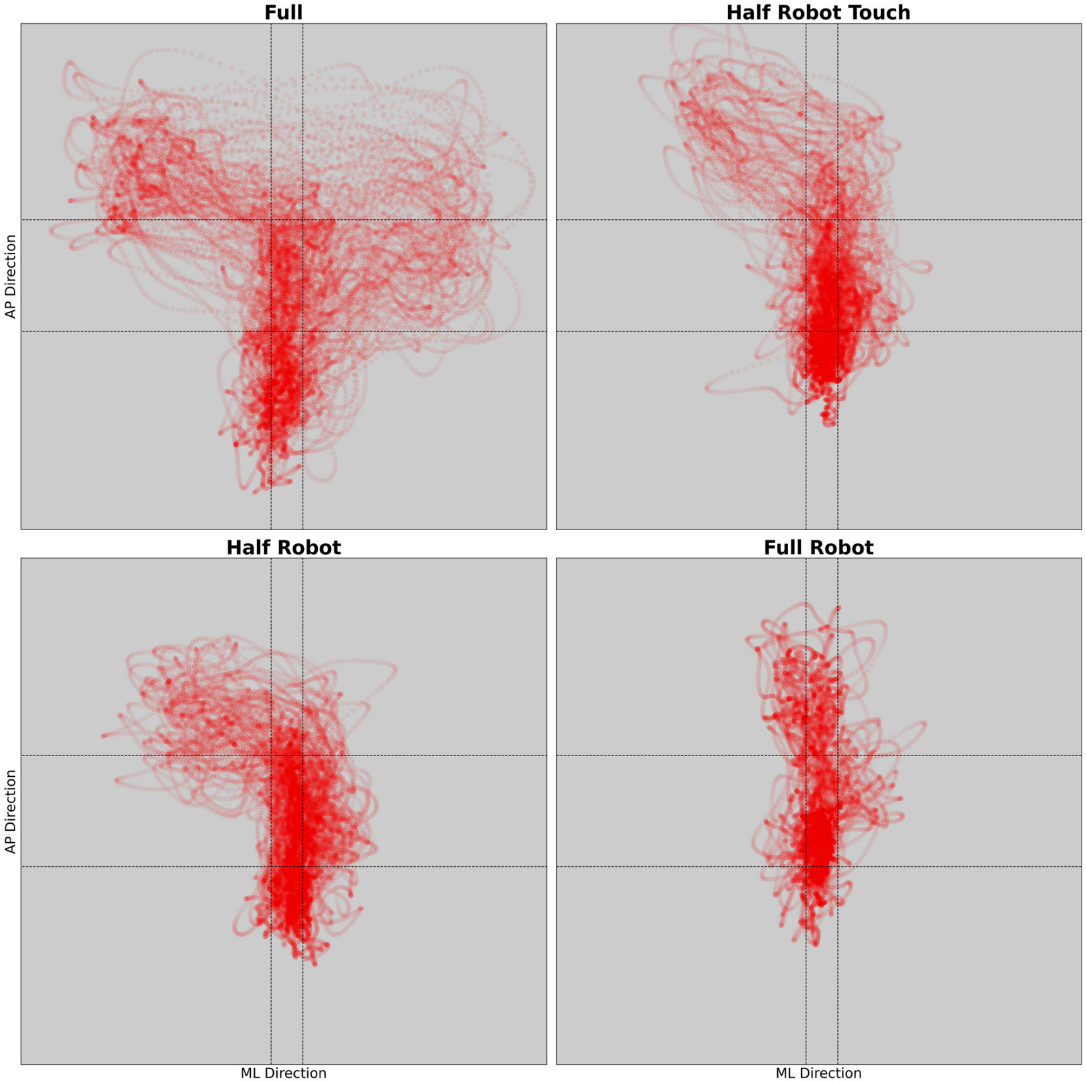
Example of the radial acceleration from the four different trials. Each dot represents a time sample. Dashed lines show the grid defining the different acceleration zones.

For each subject, a 3x3 acceleration grid has been defined by taking the 25^*th*^ and 75^*th*^ percentiles of the distribution of the acceleration values obtained from all the four trials combined. In this way, a wide central region is defined by the values of acceleration corresponding to the central portion of the grid; limit values refer to points in the other regions. An example of the acceleration values and the corresponding grid is given in Figure 2.

From this mapping, the percentage of time spent in each of the acceleration *regions* was calculated as the fraction of all the time samples falling in the *region* itself. It is hypothesized that an increase of the relative time spent in the non-central regions would be associated with a condition of increased trunk activity away from equilibrium.

#### 2.3.3. Recurrence analysis

**Figure 3:**
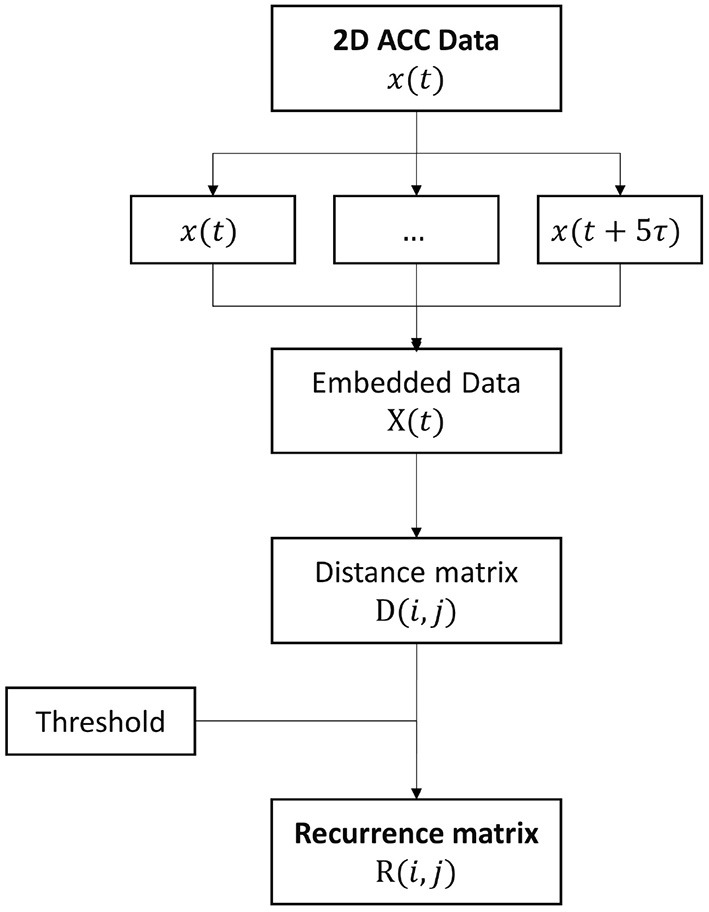
Flowchart for the embedding of acceleration data and the extraction of the recurrence matrix.

The dynamical properties of the acceleration signals have been quantified through *recurrence plots* and *recurrence quantification analysis* (RQA) (Webber and Zbilut, 1994). For RQA, the embedding dimension has been set to 5 according to Hasson et al. (2008). To investigate dynamics on a cycle-based time reference, time delay has been set to the average length of the cycle evaluated for each trial. A schematic representation of the processing steps adopted for extracting the recurrence map is given in Figure 3.

The recurrence analysis has been carried out on the radial component of the acceleration, as well as on the ML and AP components alone. Distance threshold for the definition of a recurrent point has been set to the 20% of the average pairwise distance between all points, as in Hasson et al. (2008).

From RQA analyses, three parameters were calculated as follows:

- *Recurrence (REC)*. The percentage of recurrent points (*i.e.*, the number of points in the distance matrix that are below the threshold). This is a measure of the probability of having a recurrent state (time-independent) in the system, thus higher REC reflects lower variability across repetitions.
- *Determinism (DET)*. The percentage of recurrent points belonging to a diagonal parallel to the principal one of minimum 2 points length. This is an indicator of the probability of passing through similar states in a time-dependent fashion, and higher DET values can be associated with higher predictability of movement across repetitions.
- *RATIO*. The ratio between determinism and recurrence. A high RATIO will then represent how much of the recurring patterns across repetitions is deterministic in nature.

### 2.4. Statistics

For all the parameters, a Friedman test was performed to determine the effect of the trial. The statistical significance of the differences between trials was assessed via a Wilcoxon Ranksum test, with significance set at α = 0.05 and Bonferroni correction. Effect size was characterized by means of the Cohen's *d* parameter.

## 3. Results

### 3.1. Rate of package processing

The average rate of package processing for the trial without robot collaboration was 15 ± 6 packages per minute (median ± IQR). The different robot collaboration modalities yielded rates of 8 ± 5, 12 ± 1, and 4 ± 1 packages per minute for *half robot touch, half robot*, and *full robot*, respectively.

### 3.2. Acceleration values

**Figure 4:**
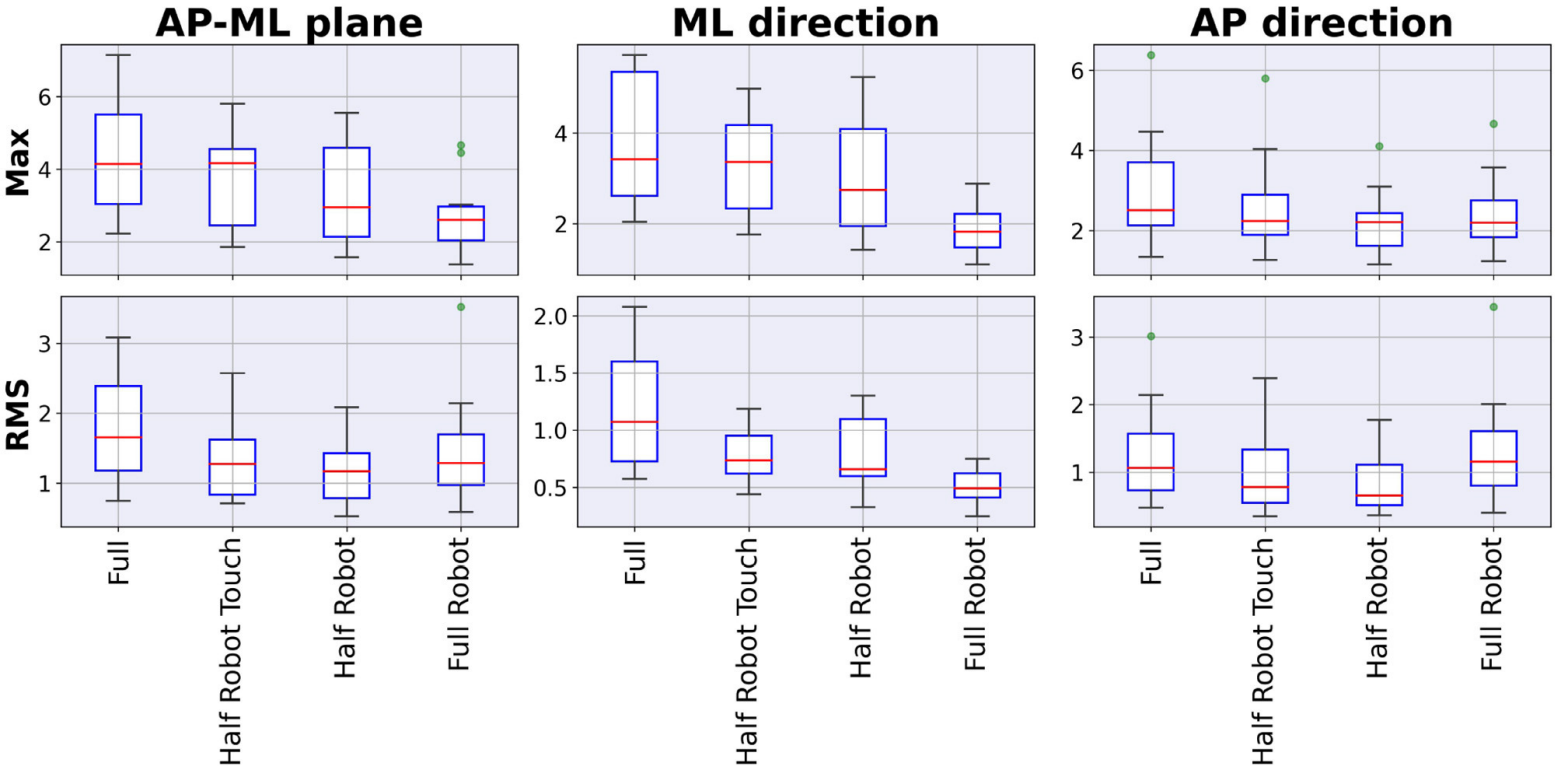
Maximum and RMS values of the acceleration recorded during the different modalities.

Peak and average acceleration values are reported in Figure 4. Values show a significant effect of the trial on all the analyzed parameters except for the RMS in the AP-ML and AP directions (maximum *p*-value, Friedman chi-squared test: 0.03.). Results show a decreasing trend of the medio-lateral acceleration values depending on the level of robot intervention (from no intervention in *full* to maximum in *full robot*) (minimum effect size for the significant differences across conditions: *d* = 1.9).

### 3.3. Phase durations

**Figure 5:**
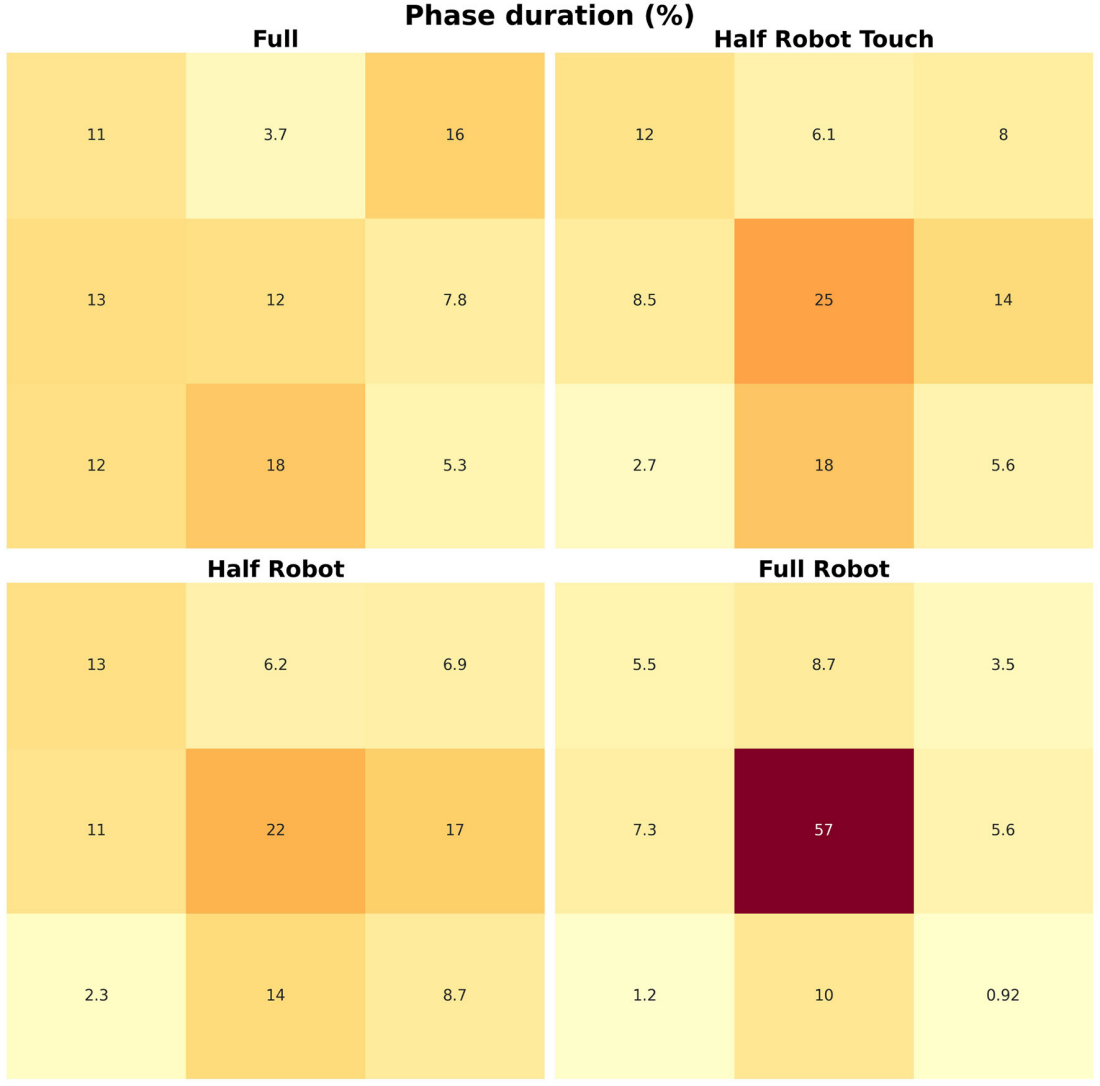
Maps for the different acceleration zones. Darker color means higher percentage of points.

Average values for the phase duration are reported in Figure 5; in this figure, the *loading* area is shown in the top right part, while the *unloading* area is shown in the top left part. The four modalities of task execution show different heat areas, representing different trunk strategies.

**Figure 6:**
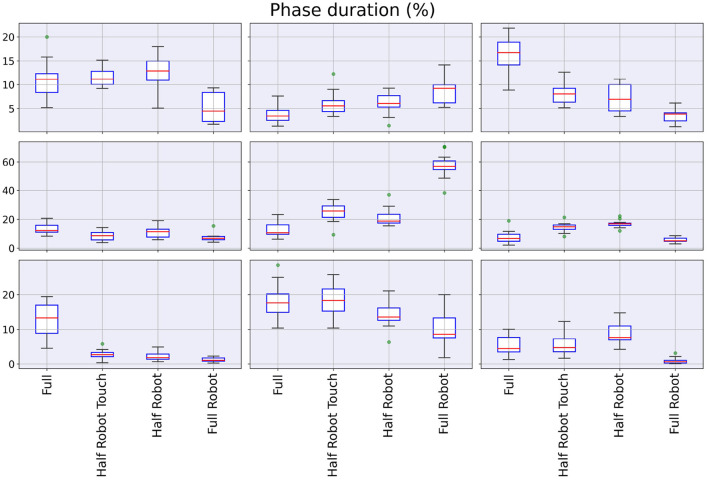
Boxplot for the values in Figure 5.

Boxplots for the distribution of the aforementioned values are reported in Figure 6. The Friedman test showed a significant effect of the trial on the time spent in all the nine acceleration *regions* (maximum *p*-value, Friedman chi-squared test: 0.02). In general, the *full robot* modality is characterized by very high percentages in the central area, with all others being more spread across the remaining regions (all *p*-values from the *post-hoc* tests below 10^−4^, minimum *d* = 0.9).

### 3.4. Recurrence maps

**Figure 7:**
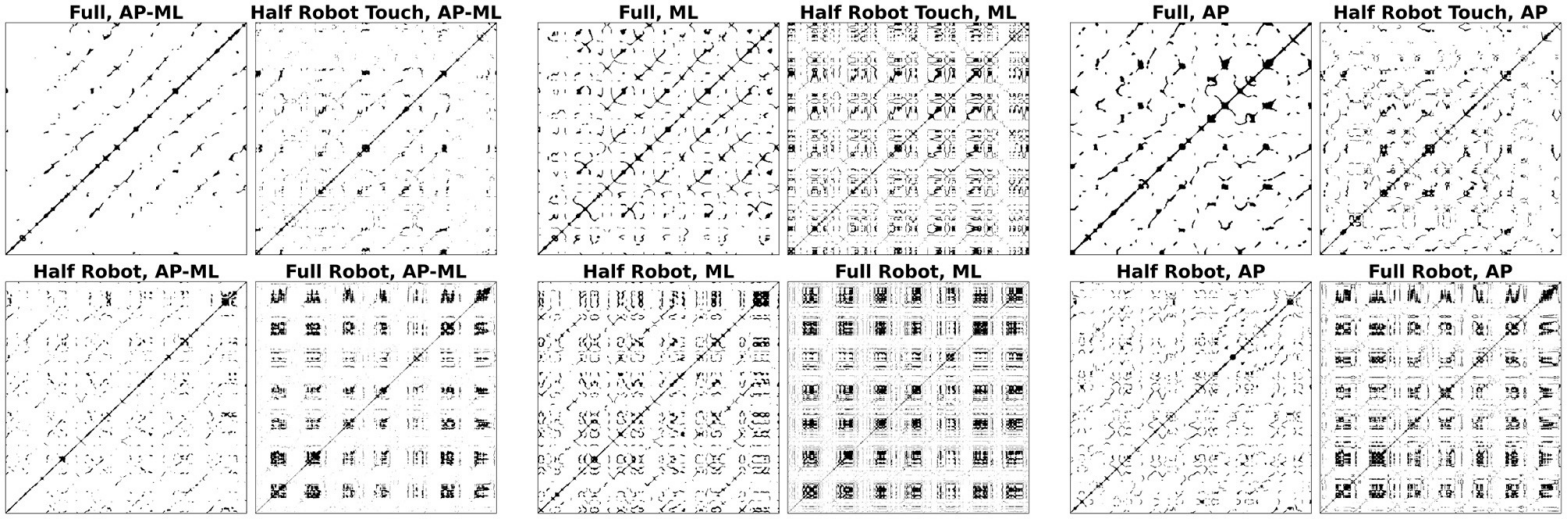
Sample recurrence maps in different trials and for different signals.

An example of the recurrence maps obtained from a subset of the cycles relative to one subject is reported in Figure 7. As a general feature, the *full robot* showed a checkered structure in all the three time series; the same structure can be also identified in the ML direction of *half robot touch*, indicating the presence of recurrent quasi-stationary states. On the contrary, the *full* modality results in a general increase of the recurrence map diagonal lines, corresponding to a higher probability of having time-dependent recurrent states (*i.e.*, a more periodical structure).

**Figure 8:**
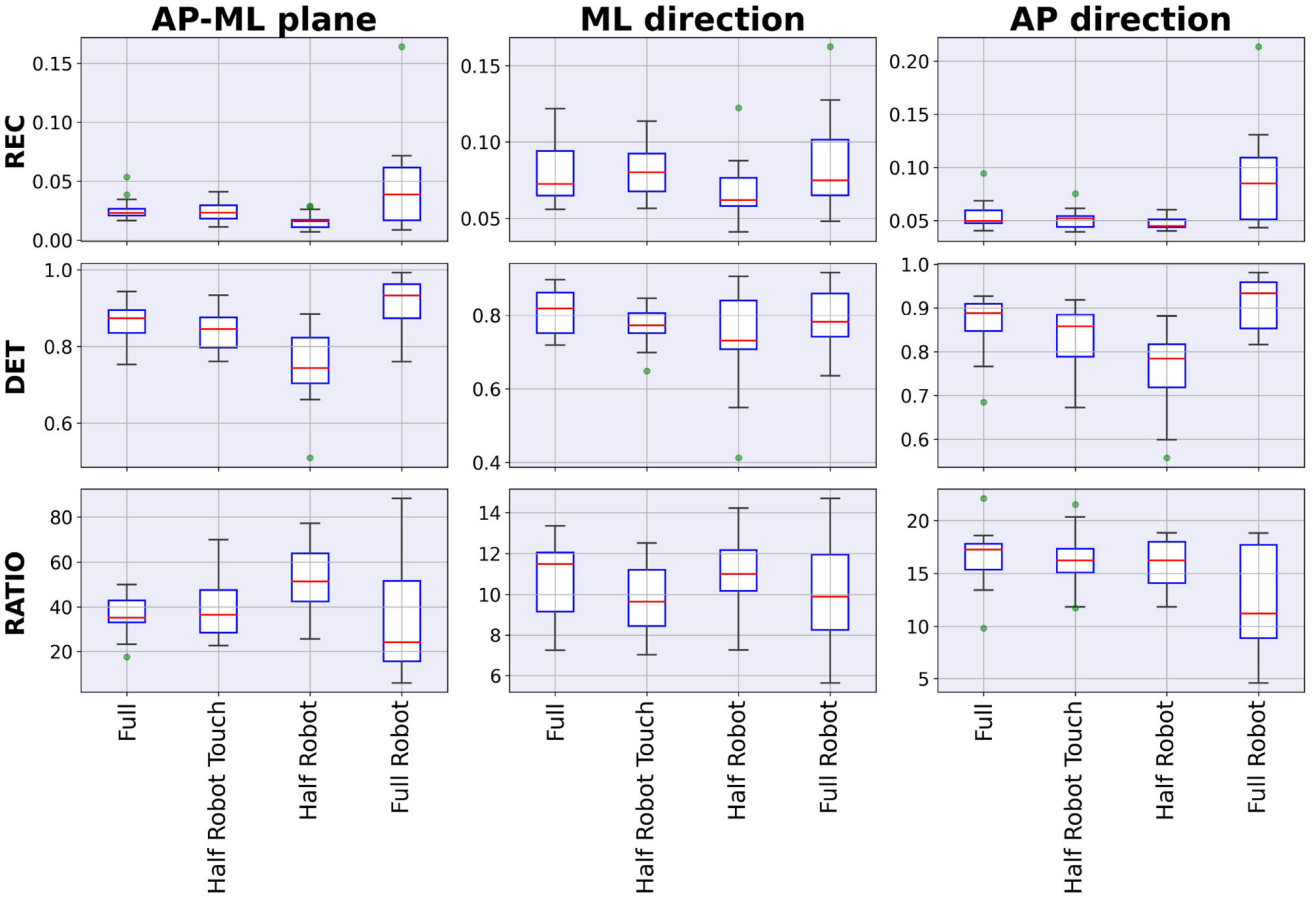
Recurrence parameters.

The values for the recurrence parameters reported in Figure 8 reflect the qualitative analysis of the sample maps shown in Figure 7. There is a significant effect of the modality on the REC and DET parameters on AP-ML and AP directions and on the RATIO parameter in the AP-ML direction (all *p*-values from the Friedman chi-squared test below 0.02); the *full* and *half robot touch* pair only showed no significant differences in terms of recurrence parameters (all *p*-values related to this *post-hoc* pair are above the significance level).

## 4. Discussion

Results presented in this study show that during a sequential human-robot collaboration activity, substantial differences in terms of trunk oscillation can be identified. In detail, different strategies for realizing the collaboration can yield different trunk movement mechanisms in terms of raw acceleration values, as well as in terms of dynamical and stability properties as quantified by recurrence quantification analysis.

In detail, the three collaboration strategies designed in our set-up aimed to test the effect of the robot intervention for most of the weight movement phases (*i.e., full robot*), in the case of very fast and subject-independent movements (*i.e., half robot*) and with a subject-defined rhythm (*i.e., half robot touch*). All these modalities give rise to different trunk movements, as described by the parameters investigated here; while maximum and RMS acceleration values suggest a lower trunk effort with an higher robot involvement, phase durations, and recurrence analysis prove that a monotonous trend cannot be identified for all the trunk oscillation features.

It is important to point out that both *full robot* and *half robot touch* include, because of the specific design of these modalities, a waiting phase; in the former case, the subject waits for the robot for most of the cycle duration, while in the latter, the waiting phase is limited to the interval between the touch start command, given by the subject to the robot, and the placement of the package to the *secondary loading area*. Despite the robot behavior was optimized to reduce these waiting times, the difference in terms of processed packages per minute is essentially driven by this specific aspect. *Full* and *half robot* modalities results to be on the two opposite sides with respect to the modality described above: the *full* modality gives rise to an optimal package handling rhythm basically due to the absence of the robot, while the *half robot* modality compels subjects to follow the robot rhythm in a strict and frenetic manner. Despite this constraint, a lower package handling rhythm identifies the *half robot* modality with respect to the *full* one, suggesting that this modality is the farthest from the optimal. Indeed, the subject seems to be in a discomfort situation, without an actual increase in the task efficiency.

The combined analysis of the maximum and RMS acceleration values show that most of the differences between modalities can be found in the ML direction. In general, the *full robot* modality, requiring the lowest trunk movements, shows lower accelerations. The effect of the waiting phase during the task execution is mostly evident in the ML RMS value, where *half robot touch* and *full robot* modalities show lower values with respect to the other two modalities. On the contrary, maximal acceleration results show that the highest value is yielded by the *full* modality, with the two *half robot* and *half robot touch* modalities being in the middle between the two limit conditions. While this behavior is to be intended only as a trend in the data (without a statistical significance), it suggests that the maximal acceleration is conditioned by the actual packages loading, action that is not performed in the *half robot touch* modality, even if subjects reach with the right hand the *loading* area to start each cycle.

The phase analysis shows that, even when no waiting phase is present (*i.e.*, during the *half robot* modality), the intervention of the robot results in a wider portion of the cycle spent in the central region of the map. On the contrary, the robot absence yields a map that is more unbalanced toward backward acceleration. The analysis of the single phase durations reported in Figure 6 shows how the *full* modality results in significantly higher values in the top-right (*i.e.*, toward the *loading* area) and bottom-left (*i.e.*, away from the *loading* area) regions, corresponding to the package loading and its movement through the scanning area. This is the only phase that is absent in all the other modalities. In addition, the two *half robot* and *half robot touch* modalities show higher values for the center-right direction of acceleration, that can be related to a different return strategy at the end of the scanning and unloading movement. Different from all the other modalities, the *full robot* modality shows very small accelerations in the ML direction: while this can be an indicator of an improved ergonomics of the movement (Granata and England, 2006), the modality is significantly slower with respect to all others and the performance, in terms of ergonomics of the movement, is not quantifiable by this information alone.

Recurrence quantification analysis proves that the four different tested modalities generate four different dynamical strategies: the *full robot* modality shows a checkered structure that indicates the alternation of different system statuses. This aspect can also be identified in the ML direction of the *half robot touch* modality: while this feature can be easily explained by the presence of the waiting phases, the *half robot touch* modality data show that this phasic behavior is realized only in the ML direction, while the AP dynamics are essentially equivalent to the ones that can be found without robot collaboration. The parameters in Figure 6 can lead to the same interpretation: this checkered structure is identifiable in slightly higher REC and DET values for both the *full robot* and *half robot touch* modalities. High DET values are also recorded for the *full* modality, suggesting that when subjects are moving without the presence of the robot, their movements are more stereotyped and matching similar states during the trial (Webber and Zbilut, 1994). In general, all the three recurrence parameters show that the *half robot touch* modality is, among the robot-assisted modalities, the one that drives the minimum perturbations from the autonomous execution of the task. Moreover, if the amount of determinism can be linked to a measure of effectiveness in task execution, the *half robot* modality seems to be not as good in these regards as the other collaboration modalities.

These results, interpreted in combination, highlight the complexity of the trunk behavior when a subject collaborates in different modalities with a robot. One main aspect of the presented results is the following: if most of the task phases are delegated to the robot, the trunk acceleration can decrease significantly, but this drives to a relevant deviation from the natural way of executing movements. This suggests that when planning the robot actions, a trade off between these two concurring aspects needs to be considered, as this may leave the subject the possibility to act as free is possible. While one of the main contributions to these results is related to the different rates associated to different modalities, results on the *half robot touch* modality prove that it is possible to leave one degree of freedom (*e.g.*, the AP direction) as naturally controlled, while exploiting a more rigid and fixed movement on the ML direction. In addition to this, during a sequential collaboration, increasing the velocity of the robot-operated phases may increase the difficulty of the task for the subject, resulting in a more chaotic movement.

Previous studies (Mancini et al., 2012) have shown that such description can be adopted for postural sway analyses in subjects with pathologies when more parameters are calculated, suggesting that the choice of a single sensor placed on the trunk can yield fine information on the subject motor control during standing posture. In this study, the choice of using simple parameters with a completely wearable set-up has been made in order to build an analytical tool that can be implemented in real time in a workplace scenario. In this sense, RQA has been carried out here by exploiting the hypothesis that this technique is able to gather information about the dynamics of the system even when a relatively short timeframe is analyzed (Webber and Zbilut, 1994).

The ergonomic implications of the presented results are still to be characterized thoroughly; however, the analyses carried out highlight important features of movement that have already been linked to the ergonomics of movement, especially in the field of postural fluctuations (Riley et al., 1999; Granata and England, 2006; Hasson et al., 2008; Mancini et al., 2012), proving that this kind of analysis yields information about the risk associated with task execution.

One of the main limitations of this analysis is associated with the strategy for HRC: no specific optimization for the robot controller was implemented to directly improve trunk movements, and this means that collaboration modalities did not change on-line based on the movement of the human operator; moreover, the interaction between the human operator and the robotic arm has been limited to discrete instants in time (*i.e.*, the handling of the load), and this might be affecting the results, generating those *switching* patterns that appeared during the collaboration modalities. Moreover, by designing the task in a purely unconstrained fashion, in terms of instruction to the operators, there is a high inter-individual variability in movement patterns, that may hinder behavior at the population level. In addition to this, there is currently no gold standard for evaluating the ergonomic performance of this kind of task, so the results that have been shown here have a descriptive meaning, and their link with direct indicators of biomechanical behavior and physical condition has to be characterized before the inclusion of such a monitoring tool in real-world scenarios.

Results discussed here should be interpreted as an indication of how to reach a sequential collaboration scenario in which the subject does not detach significantly from a natural way of performing tasks if collaboration with robots is introduced. By limiting the analyses to the acceleration profiles alone, one might conclude that the best (and possibly most effective in terms of ergonomics) way of executing the task is the *full robot* modality; however, when including RQA in the analysis, it is evident that some degree of control must be handled directly by the human operator to reach movement patterns and strategies that show the same dynamical properties as in the *full* modality.

## Data availability statement

The original contributions presented in the study are included in the article/supplementary material, further inquiries can be directed to the corresponding author.

## Ethics statement

The studies involving human participants were reviewed and approved by Ethics Committee Roma Tre University. The patients/participants provided their written informed consent to participate in this study.

## Author contributions

Conceptualization: SR, DB, MS, and SC. Methodology, software, formal analysis, writing—original draft, and visualization: SR. Validation and data curation: SR, GC, and DB. Resources and funding acquisition: SC. Writing—review and editing: SR, GC, DB, MS, and SC. Supervision and project administration: MS and SC. All authors contributed to the article and approved the submitted version.
